# Awareness of Seafood Safety Concerning Radioactivity: Country-Based Comparisons of Food Safety Issues

**DOI:** 10.3390/foods14040665

**Published:** 2025-02-15

**Authors:** Min-Sook Kyung, Hyun-Wook Do, Jung-Woon Seo, Won-Wi Moon, Sunny Ham

**Affiliations:** 1 Department of Food & Nutrition, Yonsei University, Seoul 03722, Republic of Korea; rudalstnr@naver.com (M.-S.K.); ivetta24@yonsei.ac.kr (W.-W.M.); 2 Department of Korean Cuisine, Jeonju University, Jeonju 55069, Republic of Korea; hw.do@jj.ac.kr; 3 Department of Hotel and Foodservice Management, Cheongju University, Cheongju 28503, Republic of Korea; hosecom8@gmail.com

**Keywords:** seafood, awareness, radiation, diseases, food safety

## Abstract

The present study examined how consumer awareness of seafood safety in different countries influences their concerns about radioactivity in seafood, focusing on the moderating impact of interest in food safety issues. Data from the 2021 Food Consumption Behavior Survey conducted by the 2021 Korea Rural Economic Institute were analyzed. The survey targeted 3318 primary food purchasers in households with members aged 19–75 years old. Lower safety awareness of Japanese seafood and European seafood correlated with higher levels of concern about radioactivity. Additionally, regarding the moderating effect of interest in food safety issues, lower safety awareness of European seafood correlated with greater concern about radioactivity. However, a positive moderating effect of interest in food safety issues was observed for the interaction between safety awareness of European seafood and concerns about radioactivity in seafood. The findings suggest that consumers’ safety awareness about seafood was associated with their concerns about radioactivity in seafood. Thus, it is necessary to reduce consumers’ concerns about radioactivity and elevate their safety awareness of seafood. The strategies would be suggested to elevate consumers’ perception of seafood safety awareness. The seafood industry invests in the production process to enhance the safety of seafood. The government applies new technologies to analyze seafood safety and radioactivity levels in seafood, which, thus, assures consumers’ seafood consumption.

## 1. Introduction

In 2011, the Fukushima Daiichi Nuclear Power Plant (NPS) accident in Japan led to the discharge of radioactive contaminated water into the sea, raising concerns about the contamination of seafood [1,2]. Since then, the Japanese government has implemented a thorough inspection of all Japanese seafood and the blocking of radioactive materials, but public anxiety still remains [3]. The International Atomic Energy Agency (IAEA) reported that contaminated water could travel across the Pacific Ocean and affect marine ecosystems and seafood harvested in neighboring countries [4].

According to data from the National Assembly Research Service, it is predicted that the impact of the discharge of contaminated water from Japanese nuclear power plants on Korean seafood is limited (National Assembly Research Service). In addition, radioactivity tests conducted after the discharge of contaminated water announced by the Korean government (Ministry of Oceans and Fisheries) (as of August 2024) reported that the radioactivity concentrations in popular seafood, such as cutlass fish, mackerel, and seaweed, were all appropriate (the radioactivity standards for seafood are for iodine and cesium (all less than 100 Becquerel (Bq/kg)) [5]. The US FDA reported that the risk to consumers is very low through continuous radiation testing [6]. The EU strengthened radiation tolerance standards and restricted imports after the Fukushima nuclear power plant accident in 2011 but announced that it would lift restrictions on imports of Fukushima food in July 2023 [7]. In this way, each country is trying to alleviate concerns about food safety issues by suggesting measures through strengthening standards and monitoring to suggest countermeasures for food safety issues such as radioactivity [8,9].

These cases can be used as an important background to explain the impact of radiological accidents on global food safety and consumer trust. This suggests that concerns about radioactivity are not simply a regional problem but a core issue related to food safety and that it is important to develop policies and communication strategies to secure public trust [8]. In summary, it is important to continuously monitor the presence of radioactive contamination in seafood and make efforts to mitigate potential risks by disclosing information about this.

However, despite the above efforts, domestic consumers’ concerns about seafood have significantly increased due to the release of radioactively contaminated water in Japan in 2011. This led to a consumption pattern among domestic consumers that involved avoiding Japanese seafood, particularly fresh pollack, and sea bream [10]. This trend has resulted in a reduction in the consumption of both Japanese and Korean seafood, leading to a contraction in the seafood consumption market [11].

Meanwhile, looking at the consumption of seafood, Asian countries consume more than 50% of the world’s seafood, with shrimp and tuna being the most popular types [12]. European countries prefer shellfish, and North America prefers salmon and shrimp [13]. In a study of 319 Korean consumers, 62% were consumers of fish (mackerel, cutlass fish, flounder, rockfish, salmon, tuna, etc.), 17.2% of seaweeds (brown seaweed, kelp, seaweed, etc.), and 8.5% of mollusks (octopus, squid, etc.), shellfish (clams, scallops, oysters, etc.), and crustaceans (crabs, shrimp, etc.), each accounting for 3.8%. Mainly fish and seaweed were found to be highly preferred [14]. Seafood provides essential nutrients, such as omega-3 fatty acids, vitamins, and minerals [15], and is also delicious, making it one of the foods highly preferred by consumers.

However, consumers are concerned about the contamination of seafood due to radiation exposure, and the greatest concern is likely to be related to disease problems resulting from this. In fact, radiation-related health risks, such as cancer, genetic disorders, and other physical conditions, have been extensively documented following nuclear disasters like Chornobyl in 1986 and Fukushima in 2011 [16,17]. To summarize, it is believed that the issue of radioactivity in seafood can cause disease-related anxiety in consumers and that this anxiety can affect consumer psychology or direct consumption of seafood.

Meanwhile, public concerns about radioactivity in seafood have increased, prompting governments to implement comprehensive measures to ensure food safety and bolster public confidence. The Korean Ministry of Food and Drug Safety (KMFDS) has banned the import of 15 types of agriculture and seafood from 27 regions, including Fukushima, and set a Cs limit of 100 Bq/kg for all imported food items [18]. The Korean Ministry of Oceans and Fisheries (KMOF) and KMFDS are the primary agencies responsible for seafood safety management. The KMOF oversees production and distribution and conducts inspections, tests, research, and examinations of both domestic and imported seafood in nationwide markets [19,20]. Recent initiatives by the Korean government offer radioactivity safety information regarding seafood through websites covering the stages of catching, storing, and selling [21]. Additionally, in response to public requests, the government website of the Public Requests for Seafood Radioactivity Inspection program displays safety information regarding seafood [22].

The KMFDS primarily manages the distribution phase by conducting documentation reviews and sensory inspections of all imported foods, including those from Japan [18]. Owing to concerns arising from the release of radioactively contaminated water, stringent inspections encompassing both precise and random testing have been applied to Japanese seafood [18]. The administration maintains a website that reports the radioactivity inspection status of all imported foods, including Japanese seafood [18], ensuring transparent dissemination of information and fostering public trust in food safety [23].

Government-level policies and communication strategies are imperative for educating the public and raising awareness of seafood safety. The KMFDS conducted the “Public Forum for Seafood Safety Management” to enhance public understanding of seafood safety management [24,25]. To summarize, the government carries out thorough safety management and provides correct information to consumers for all imported seafood, including domestic and Japanese products. This can improve consumers’ awareness of safety by country of origin, reduces concerns about radioactivity, and is expected to have a positive impact on seafood consumption by providing correct food safety information.

Regarding the safety awareness of seafood based on its country of origin, notable research findings related to seafood and radioactivity are as follows: The studies conducted by Jeong et al. (2022) [26] and Noh (2023) [27] observed a temporary decline in consumer purchases following media reports on radioactivity leaks in Japan, indicating that safety awareness, particularly concerning Japanese seafood, influence purchasing decisions. Yun and Kim (2022) [14] disclosed that apprehensions about the discharge of radioactive water from Japanese nuclear facilities affected seafood purchases, with notable concerns expressed for both domestic (85.4%) and imported (85.5%) seafood. This implies that the release of radioactivity-contaminated water negatively affected Koreans’ seafood purchasing of domestic seafood in addition to imported seafood. In summary, the media forecasting about the release of radioactivity-contaminated water resulted in a negative effect on the consumers’ safety awareness of seafood and then a decline in seafood purchase. This implies that the release of radioactively contaminated water affects the purchase of seafood irrespective of its origin.

Most research on this topic has focused on the ramifications of discharging radioactively contaminated water for the procurement of Japanese seafood. In light of the burgeoning concerns about seafood safety, particularly regarding radioactivity, it is critical to scrutinize how awareness of seafood safety, specifically radioactivity apprehension, influences consumer seafood consumption patterns. In addition, it is expected that verifying the moderating effect of interest in food safety issues in relation to the impact of safety awareness by the country of origin of the seafood on the level of concern about radioactivity will have a positive impact on the consumption of seafood.

Accordingly, this study seeks to establish and verify the following hypotheses.

**Hypothesis** **1.** *Consumers’ safety awareness of seafood has a negative effect on their level of concern about radioactivity.*

**Hypothesis** **1-1.** *Consumers’ safety awareness of domestic seafood has a negative effect on their level of concern about radioactivity.*

**Hypothesis** **1-2.** *Consumers’ safety awareness of Japanese seafood has a negative effect on their level of concern about radioactivity.*

**Hypothesis** **1-3.** *Consumers’ safety awareness of Chinese seafood has a negative effect on their level of concern about radioactivity.*

**Hypothesis** **1-4.** *Consumers’ safety awareness of European seafood has a negative effect on their level of concern about radioactivity.*

**Hypothesis** **2.** *The influence of safety awareness of seafood by country of origin (domestic/Japanese/Chinese/European) on the level of concern about radioactivity varies depending on the level of interest in food safety issues.*

**Hypothesis** **2-1.** *The influence of safety awareness of domestic seafood on the level of concern about radioactivity varies depending on the level of interest in food safety issues.*

**Hypothesis** **2-2.** *The influence of safety awareness of Japanese seafood on the level of concern about radioactivity varies depending on the level of interest in food safety issues.*

**Hypothesis** **2-3.** *The influence of safety awareness of Chinese seafood on the level of concern about radioactivity varies depending on the level of interest in food safety issues.*

**Hypothesis** **2-4.** *The influence of safety awareness of European seafood on the level of concern about radioactivity varies depending on the level of interest in food safety issues.*

Therefore, this study aimed to explore how consumers’ safety awareness of seafood from various countries affects their concerns about radioactivity and examine the moderating role of interest in food safety issues. The specific objectives were to (1) identify consumer behavior about seafood purchases; (2) assess safety awareness and interest in food safety issues about seafood based on the country of origin; (3) examine concerns about hazards linked to seafood consumption; and (4) investigate the impact of seafood safety awareness based on the country of origin on radioactivity concerns, considering the moderating effects of interest in food safety issues.

## 2. Materials and Methods

### 2.1. Research Procedure

**Figure 1:**
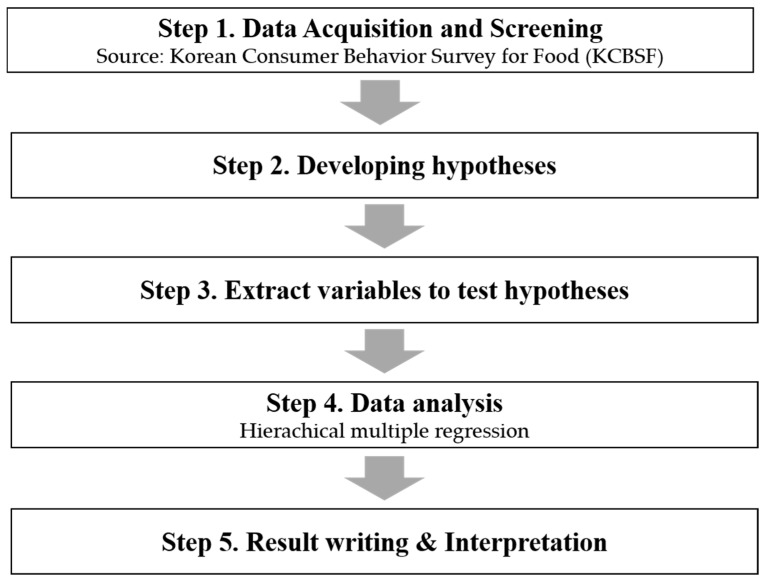
Research procedure.

The research procedure was conducted in five steps (Figure 1). First, for this study, secondary data were acquired, which were collected from the “Korea Food Consumer Behavior Survey (KCBSF)” conducted by the Korea Rural Economic Institute (KREI). The data were collected from 6355 people aged 19 to 75 years old. Among them, 3318 primary food purchasers were selected as research subjects for this study (Step 1).

Second, a total of eight research hypotheses were developed to determine how consumers’ safety awareness of seafood from various countries affects their concerns about radioactivity and to investigate the moderating role of food safety interests (Step 2).

Third, variables to investigate the study objectives were extracted from the “Korean Consumer Behavior Survey for Food (KCBSF)” research instrument. The main variables selected in this study were 1 question about levels of concern regarding radioactivity in seafood, 4 questions about safety awareness of seafood based on the country of origin (Korea [domestic]/Japan/China/ European Union (EU)), 1 question about interest in food safety issues, 3 questions about consumer behavior regarding seafood purchases by primary food purchasers, 8 questions about demographic/socioeconomic variables, and 2 questions about health factors (Step 3).

Fourth, hierarchical multiple regressions were applied to empirically test the proposed research hypotheses (Step 4).

The last was writing and interpreting the outcomes from the statistical analysis on the causal relationship between the safety awareness of seafood, based on the country of origin, and the levels of concern regarding radioactivity in seafood. It also explored the moderating effect of interest in food safety (Step 5).

### 2.2. Data Collection

Data were obtained from the “Korean Consumer Behavior Survey for Food (KCBSF)” conducted by the Korea Rural Economic Institute (KREI) [28]. Since 2013, the KREI has administered the KCBSF annually to augment food supplier competitiveness and consumer satisfaction [29]. Basic research was conducted from September 2012 to March 2013 to conduct a preliminary review of the survey questions and methods of the “Korean Consumer Behavior Survey for Food (KCBSF)” [29].

Out of 6355 respondents, the cohort for this study comprised 3318 adults aged 19–75 years who were the principal food purchasers within their households [29]. A combination of samples independently extracted using temporal juncture and colony-type sampling was used to select a representative cross-section of Korean consumers [29]. The temporal sampling involved substituting the 2013 sample design with a counting district linked to 2018 [29]. For the colony-type sampling, selections were made from the population census collection district and the Kook Min Bank new apartment list according to the 2016 sample design [29].

Data collection involved personal interviews following the acquisition of consent for personal information usage from the participants [29]. The survey was conducted from 25 May to 6 August 2021 [29].

After obtaining the data, 3875 responses were screened. Subsequently, 3318 responses were deemed suitable for the final analysis after excluding non-responses (172), ineligible responses (85), and others (300) [29]. Secondary data analyses were approved by the University Institutional Review Board (7001988-202310-HR-2078-01E), with an exemption form (containing no personally identifiable information about the sample).

### 2.3. Research Instrument

The participants were the main purchasers of food and food ingredients in their households. The questionnaire for these primary purchasers included questions regarding food purchasing and consumption behaviors, food waste discharge and reduction efforts, specific food purchase and consumption patterns, dining out habits, eating habits, lifestyle, and household demographics [30].

This study selected consumer concern regarding radioactivity in seafood as the dependent variable and awareness of seafood safety based on the country of origin, which seemed to be the most relevant factors that could affect this. In addition, interest in food safety issues was selected because it seemed to be a necessary control variable for establishing a promotional strategy for food safety. Meanwhile, because this study was conducted on consumers, household or respondent demographic, socioeconomic, and health factors could be variables highly related to safe food purchases and were presented as independent and control variables. There may be many other variables, but the two most appropriate variables related to food safety awareness in secondary data called food consumption behavior survey questions are safety awareness by the country of origin and interest in food safety issues.

The questions analyzed in this study comprised four parts: consumer concern regarding radioactivity in seafood, awareness of seafood safety, interest in food safety issues, and demographic, socioeconomic, and health factors.

The first part of the questionnaire addressed the levels of concern regarding radioactivity in seafood, which served as the dependent variable in this study. To ascertain this, participants were asked, “How concerned are you about the hazards of food?” Specifically, concern about radioactivity in seafood was gauged using a Likert scale ranging from 1 point (“I am not at all concerned”) to 5 points (“I am very concerned”).

The second part of the questionnaire contained four questions about awareness of seafood safety based on the country of origin (Korea [domestic]/Japan/China/European Union (EU)), which were used as independent variables. To evaluate this, the question posed was “What do you think about the safety of imported foods?” The perceived safety of seafood from Korea, Japan, China, and the EU was measured using a Likert scale ranging from 1 (“not safe at all”) to 5 (“very safe”).

The third part of the questionnaire concerned interest in food safety issues and was used as a control variable. Participants were asked, “How interested are you in food safety issues in general?” Responses were quantified on a 5-point Likert scale ranging from 1 (“not at all interested”) to 5 (“very interested”).

The fourth part of the questionnaire assessed consumer behavior regarding seafood purchases by primary food purchasers using three questions. (1) The primary criteria considered when purchasing seafood were identified from options, such as price, taste, safety, quality, nutrition (health), purchase convenience, and cooking convenience. (2) When purchasing seafood, consumers verified information such as price, production area name (e.g., West Coast), country of origin (e.g., domestic and Chinese), freshness, brand (e.g., Chambada and Haneultteul), appearance (shape and size), promotional status (seller recommendation or event product), variety, packaging conditions, other certifications (e.g., hazard analysis critical control point), and added functionality. (3) The frequency of purchasing various seafood categories, such as fisheries products, seaweeds (e.g., kelp and nori), shellfish, mollusks (e.g., squid and octopus), and crustaceans (e.g., snow crab), was measured on a 6-point Likert scale ranging from 1 (“less than once a month”) to 6 (“every day”).

The fifth part of the questionnaire addressed household or respondent demographic, socioeconomic, and health factors. (1) The demographic and socioeconomic variables included sex, age, educational level, occupation, average monthly household income, household size, presence of adolescent members, and administrative district (dong/eup/myeon). (2) The health variables were evaluated on a 5-point Likert scale based on responses to two questions regarding interest in health and current health status.

### 2.4. Data Analysis

The data collected in this study were analyzed using SPSS 26.0 for Windows (IBM, Armonk, NY, USA). Given that the KCBSF was conducted using a complex sampling design, the analysis was performed using a weighted sum approach, in which individual respondents’ survey values were multiplied by the sample weight [29]. Accordingly, a complex sample analysis plan file was created to reflect the strata, clusters, and weights [29], in line with the characteristics of the survey data.

The analysis methods employed were twofold.

1. Descriptive analysis: Using complex sample frequencies and descriptions, demographic and socioeconomic characteristics, health characteristics, consumer behaviors related to seafood purchase, safety awareness, and food safety based on the country of origin of the seafood were delineated. Additionally, the levels of interest in food safety issues and concerns regarding seafood hazards were evaluated.
2. Hierarchical multiple regression analysis: A hierarchical multiple regression analysis using a complex sample general linear model was conducted to examine (1) the impact of safety awareness based on the country of origin on concerns about seafood hazards and (2) the moderating effect of interest on food safety issues. To address potential multicollinearity when analyzing the moderating effect, an interaction term was generated through the mean centering of the independent and control variables before the final analysis. Covariates were controlled by sequentially entering demographic, socioeconomic, and health variables into each model. Subsequently, the independent variable (seafood safety awareness based on the country of origin), control variable (interest in food safety issues), and interaction terms were incorporated for further analysis. Hierarchical multiple regression analysis is a necessary analysis method to analyze the impact of safety awareness by the country of origin of seafood on the level of concern about radioactivity in seafood and the controlling effect of interest in food safety issues [31]. However, there may be limitations, such as loss of sensitivity in multivariate analysis due to data weighting. This study used hierarchical multiple regression analysis to verify causal relationships by gradually introducing variables, and it presented an efficient estimator for the impact on the dependent variable by considering the correlation between variables.

## 3. Results

### 3.1. Respondent Profiles

Table 1 summarizes the respondents’ profiles. Of the 3318 participants, a significant number (82.2%) were women, which is because this study was conducted on those who mainly purchase food in the family. The age distribution was skewed toward older adults, with 32.7% aged 60 years or above, followed by those in their 50s (21.7%), 40s (21.6%), 30s (18.4%), and 20s (5.6%). Regarding educational attainment, 58.6% completed high school or lower, whereas 41.4% attained a college degree or higher. Most respondents were employed in management, office, or professional jobs (29.5%), and 21.3% were housewives. The monthly household incomes varied, with the largest group earning less than KRW 2 million (21.3%), followed by the income brackets of KRW 2 million to 3 million (20.9%) and KRW 3 million to 4 million (15.8%). The high-income bracket of KRW 6 million or more accounted for 15.5% of the participants, with the remaining groups earning between KRW 4 million and KRW 6 million. Households predominantly consisted of one-person households (35.6%), two-person households (27.7%), three-person households (18.3%), and households with four or more persons (18.4%). Most households included adolescent members (86.8%), whereas the remainder did not (13.2%). Most respondents resided in urban areas (80.1%), whereas the rest lived in rural regions, such as myeons/eups (19.9%).

### 3.2. Health- or Diet-Related Characteristics of Respondents

Table 2 presents the respondents’ health characteristics. On average, their interest in health scored 3.77 out of 5 points, and their self-assessed current health status averaged 3.62 points.

### 3.3. Consumer Behaviors Related to Purchasing Seafood

Table 3 lists the consumer behaviors related to seafood purchases. “Quality” (29.6%) was the most important selection criterion when purchasing seafood, followed by “taste” (29.4%) and “safety” (13.2%). Additionally, “nutrition (health)”, “convenience of purchase”, and “convenience of cooking” were selection criteria used by less than 10% of the participants. Among the information checked when purchasing seafood, “freshness” was the highest at 47.4%, followed by “country of origin (domestic, China, etc.)” at 15.7%, and “name of production area (West Coast, etc.)” at 11.6%.

### 3.4. Frequency of Purchasing Seafood

**Figure 2:**
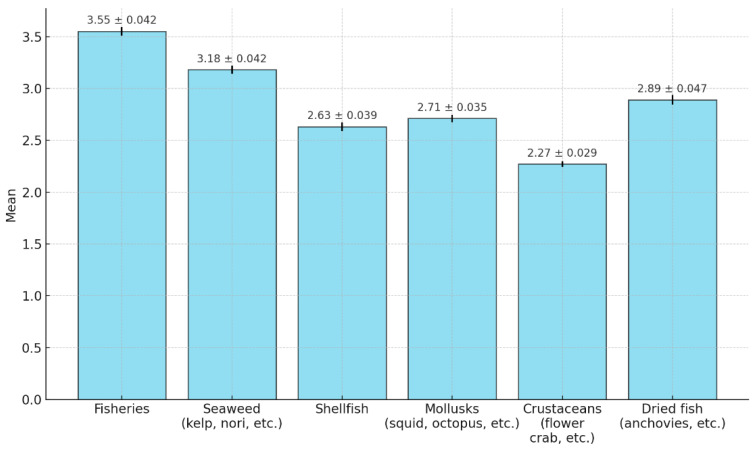
Frequency of purchasing seafood (N = 3318).

The frequency of purchasing seafood was analyzed on a 6-point scale, with “every day” representing the highest frequency and “less than once a month” representing the lowest frequency. A higher score indicated a higher frequency of purchase (Figure 2; Table 4). Fish showed the highest purchasing frequency at 3.55 points out of 6, followed by seaweed (kelp, nori, etc.; 3.18 points), dried fish (anchovies, etc.; 2.89 points), mollusks (squid, octopus, etc.; 2.71 points), shellfish (2.63 points), and crustaceans (flower crab, etc.; 2.27 points).

### 3.5. Safety Awareness of Seafood Based on Country of Origin

**Figure 3:**
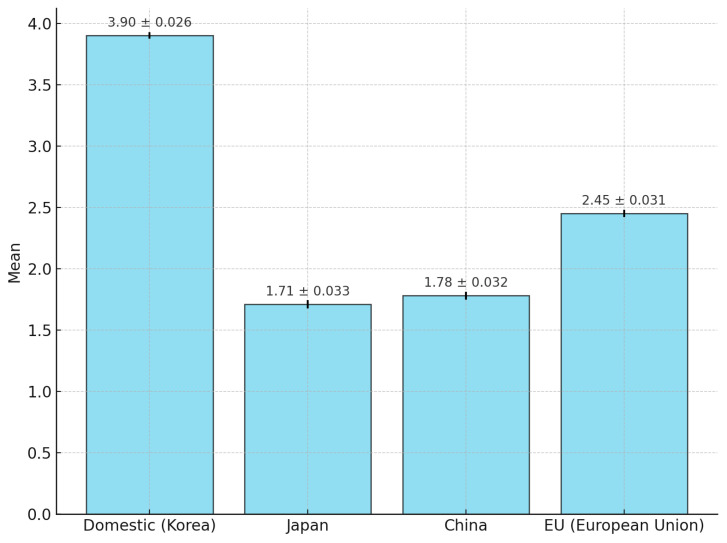
Safety awareness of seafood based on country of origin (N = 3318).

Figure 3 and Table 5 present the safety awareness of seafood according to the respondents’ country of origin. Awareness of Korean seafood was the highest at 3.90 points out of 5, followed by European seafood at 2.45 points and Chinese seafood at 1.78 points. The level of awareness of Japanese seafood was lowest at 1.71 points.

### 3.6. Concerns About Hazards in Seafood

Table 6 displays respondents’ concerns regarding seafood hazards. “Radioactivity” showed the highest level of concern with 4.23 points (out of 5 points), followed by “heavy metals, environmental hormones, etc.” with 4.18 points, “food poisoning bacteria” with 4.15 points, “fish antibiotics” with 3.91 points, “natural toxins (puffer fish, etc.)” with 3.88 points, and “allergens” with 3.80 points. “Foreign substances” indicated the lowest level of concern with 3.67 points. The overall average hazard for seafood was 4.09 out of 5, indicating a high level of concern.

### 3.7. Moderating Effect of Interest in Food Safety Issues on Relationship Between Seafood Safety Awareness Based on Its Country of Origin and Concerns About Radioactivity

A multiple regression analysis was conducted to verify the moderating effect of interest in food safety issues on the relationship between seafood safety awareness based on the country of origin and concerns about radioactivity. Table 7 presents the results of this analysis.

In Model I, demographic and socioeconomic characteristics and health variables were included as control variables along with the independent variables of interest. The explanatory power of Model I was 9.4%. Concerns about radioactivity were greater among respondents in their 30s (B = 0.209) (*p* < 0.05) and 50s (B = 0.164) (*p* < 0.05) than those aged 60 or older. Concerns about radioactivity were greater when the average monthly household income was less than KRW 2–3 million than when it was more than KRW 6 million (B = 0.250) (*p* < 0.05). Additionally, households with three members (B = 0.214) (*p* < 0.05) showed greater concern about radioactivity than those with four or more members. Furthermore, a higher level of interest in health (B = 0.108) (*p* < 0.001) correlated with a higher level of concern about radioactivity. Meanwhile, lower safety awareness of Japanese (B = −0.127) (*p* < 0.01) and Chinese seafood (B = −0.111) (*p* < 0.05) correlated with increased concern about radioactivity.

The explanatory power of Model II was 9.6%. In Model II, interest in food safety issues was used as a control variable. However, it did not have a statistically significant effect on the level of concern about radioactivity.

In Model III, the interaction term was used. The explanatory power of Model III was 10.1%. In this model, interest in food safety issues significantly moderated the relationship between awareness of seafood safety based on the country of origin and concerns about radioactivity. Among the places of seafood origin, the effect of safety awareness of European seafood on the level of radioactivity concerns varied significantly depending on the level of interest in food safety issues (B = 0.077) (*p* < 0.05). Specifically, lower safety awareness of European seafood correlated with a higher level of concern about radioactivity (B = −0.068) (*p* < 0.05). Similarly, the interaction between interest in food safety issues and safety awareness of European seafood showed a positive moderating effect (B = 0.077) (*p* < 0.05). This indicated that the control variable buffered the influence of the independent variable on the dependent variable [32]. These results revealed a significant moderating role of interest in food safety issues in the relationship between seafood safety awareness and concerns about radioactivity.

Hypothesis 1-1 was not supported. That is, consumers did not relate the safety of domestic seafood to the level of concern about radioactivity. Hypotheses 1-2, 1-3, and 1-4 were supported, which indicated consumers’ lower safety awareness was related to their higher concerns about radioactivity in Japanese, Chinese, and European seafood. For Hypothesis 2, only Hypothesis 2-4 was supported, while Hypotheses 2-1, 2-2, and 2-3 were not supported. That is, consumers’ interest in food safety issues had a positive influence on the interaction between their safety awareness and European seafood and concerns about radioactivity in European seafood.

## 4. Discussion

The findings of this study can be summarized. While consumers showed serious concerns about radioactivity in seafood, for Japanese and European seafood safety awareness, consumers showed a negative relationship with radioactivity concerns. Furthermore, only for European seafood safety awareness, consumers’ interest in food safety was positively significant to the relationship with radioactivity concerns. The summary postulates that consumers perceive radioactivity concerns seriously. In particular, when consumers sense Japanese or European seafood with lower safety, they tend to have higher radioactivity concerns. For consumers who have stronger interests in food safety issues, the lower the safety awareness of European seafood, the higher the radioactivity concerns. The following recommendations emerge from these findings.

First, consumer behavior in seafood purchasing was examined, including selection criteria, information sought at purchase, and purchase frequency. This analysis indicates that consumers prioritized quality, taste, and safety when purchasing seafood. Freshness emerged as the paramount information considered, followed by country of origin. These results align with those of previous studies, suggesting that quality and safety are critical factors influencing seafood-purchasing decisions [33]. Purchase frequency varied across types, with fish, seaweed (such as kelp and nori), and dried fish (such as anchovies) being the most common. Additionally, Park (2017) [34] found that fish products constituted the largest share (46.9%) of purchases at the Suhyup shopping mall, underscoring the significance of seafood in consumer buying behavior (refer to Table 3 and Table 4 and Figure 2).

Second, an analysis of safety awareness based on the seafood’s country of origin revealed a ranking order of Korea, the EU, China, and Japan. Notably, Chinese and Japanese seafood were perceived as less safe, scoring below two on the five-point scale. This finding is consistent with the existing literature, suggesting that Korean seafood is perceived as safer than imported seafood [35]. Negative sentiments toward imported Japanese seafood have led to heightened risk awareness and influenced purchasing behaviors [36]. Among food safety awareness items, the belief that domestic products are safer than imported products received the highest level of agreement in a previous study [37]. Similarly, the Korean Consumer Safety Sentiment Index indicates that imported seafood has the lowest perceived safety [38] (refer to Table 5 and Figure 3).

Third, consumer concerns about seafood-related hazards were ranked, in descending order, as follows: radioactivity, heavy metals, environmental hormones, and food poisoning bacteria. The average score for seven items concerning hazardous factors in seafood was notably high (4.09 out of 5). This finding is consistent with research indicating that food safety concerns (39.1%) surpass societal safety concerns (34.5%) [39]. A previous study on the keyword analysis of hazards highlighted a spike in mentions of radioactivity in 2011 and 2013, reflecting the influence of the Fukushima nuclear incident [39]. Nuclear power plant accidents have led to global radioactive leaks. Examples include the 1957 Mayak Nuclear Fuel Reprocessing Plant accident in the Soviet Union, the 1979 Three Mile Island Nuclear Power Station accident in the United States, the 1986 Chornobyl Nuclear Power Station accident in the Soviet Union, and the 2011 Fukushima Daiichi Nuclear Power Plant accident in Japan [40]. The 1986 Chornobyl disaster heightened international concerns regarding nuclear safety. Consequently, on 24 October 1996, the IAEA ratified the Convention on Nuclear Safety [41,42], which characterized a radioactivity release incident as an event with substantial consequences for people, the environment, and the facility itself [43] (refer to Table 6).

Fourth, in the examination of the relationship between consumers’ seafood safety awareness and radioactivity concerns by country of origin, Japanese and European seafood were significant and negative (B = −0.145; B = −0.068). That is, the lower consumers’ safety awareness of Japanese or European seafood, the higher their concerns were about radioactivity. This implies that consumers seriously perceive Japanese or European seafood safety, and that is related to their radioactivity concerns. This finding indicates that governments should make strong efforts to disseminate accurate and objective information about Japanese and European seafood safety (refer to Table 7).

Heo et al. (2020) [44] identified social, demographic, and subjective perceptions, experiences, and trustworthiness as factors influencing seafood safety trust. Their findings, which indicated higher levels of trust among women, older individuals, and those valuing health benefits, are consistent with the identified factors influencing radioactivity levels. Jeong et al. (2022) [26] and Noh (2023) [27] observed a decline in Japanese seafood purchases following a radioactive water leak. Kang (2015) [35] also noted a significant reduction in seafood consumption owing to the 2011 Japanese nuclear incident. These studies indicated a diminishing awareness of seafood safety in Japan. Furthermore, as evidenced in this study, heightened consumer anxiety regarding radioactivity influences consumption patterns. In this study, the explanatory power in the final selected model for the moderating effect of interest in food safety issues in the relationship between awareness of the safety of seafood by place of origin and concern about radioactivity was found to be 10.1%. This was found to be similar to the studies of Jin et al. (2014) [45] (12%) and Yoon et al. (2013) [46] (5.3%). Cohen, J. (1988) [47] stated that in social science research, such as surveys, if the coefficient of determination is 2% or more, it has small explanatory power, and if it is 13% or more, it has medium explanatory power. Based on the literature and previous studies, the explanatory power of this study falls in the acceptable range (refer to Table 7).

Additional research results in this regard are as follows. Lee et al. (2021) [48] indicated that radioactivity levels in domestically distributed seafood are within safe limits. Gwak et al. (2015) [49] found no detectable radioactivity in processed seafood products, and Kim et al. (2015) [50] reported the absence of radioactive contamination in domestically distributed seafood from 2013 to 2015. Despite ongoing consumer concerns regarding radioactivity, these studies collectively affirm that distributed food products are free of detectable radioactivity, underscoring the efficacy of the government’s comprehensive management system for seafood from production to distribution. Heo et al. (2020) [44] advocated the active use of quality certifications, such as the Seafood Quality Certification System and the Seafood Traceability Management System, to manage seafood safety. They also emphasized the importance of promoting these systems to consumers [44]. Kang (2015) [35] found that 47.4% of respondents would increase their seafood consumption if accurate safety information was provided. Kang et al.’s (2013) [51] study found that the group with a high level of food safety awareness and practice had the highest level of interest in and understanding of dietary information. It can be seen that increasing interest in food safety can influence desirable consumption considering food safety. To summarize, this underscores the need for improved seafood safety communication to foster an overall interest in food safety issues and solutions.

This study is also unique in examining consumers’ interests in food safety issues as a moderating factor. The analysis found that only for European seafood, consumers’ food safety interests served as a positive moderator (B = 0.077). While European seafood safety awareness negatively affected radioactivity concerns, interest in food safety acted as a positive moderator. That is, while consumers perceived European seafood safety as lower, they tended to show radioactivity concerns more, and their food safety interests made the relationship stronger. When consumers had higher interests in food safety issues, they tended to have a more negative relationship between their seafood safety awareness and radioactivity concerns.

These findings highlight the potential need for effective communication and promotion of seafood safety information, particularly for European-sourced products, to mitigate concerns about radioactivity and bolster consumer trust. The Korea Institute for Health and Social Affairs and the Korea Maritime Institute both emphasize the government’s role in reducing anxiety through accurate information dissemination and improved labeling systems [39,52]. This aligns with the results of this study, in which interest in food safety issues moderated the impact of European seafood safety awareness on radioactivity concerns. Ultimately, fostering greater interest in food safety issues may address the low safety awareness of imported seafood, reduce radioactivity-related anxiety, and encourage seafood consumption. In particular, the results of this study show that it is important to increase interest in food safety issues in order to resolve the negative image of increasing concerns about radioactivity.

Awareness of safety varies depending on the country of origin of seafood, and in the case of Korea, this can be seen to vary depending on whether it is frequently encountered in the country or difficult to encounter. In the case of shrimp, squid, and mackerel, imported seafood is chosen because its prices are lower than domestic seafood [53]. In addition, salmon, lobster, and bluefin tuna are seafood that are difficult to find in Korea, so consumers choose imported seafood, and among imported seafood, they appear to prioritize freshness and safety [53]. In other words, the price of imported seafood is lower than that of domestic seafood, which means that awareness of safety is also lower. In the case of seafood that is difficult to access in Korea, it can be seen that there is a tendency to select imported seafood considering safety. In other words, food safety can be an important consideration for both domestic and imported seafood. Therefore, the seafood industry should invest in the production process to enhance the safety of seafood. Furthermore, in order to increase interest in food safety issues, it is important to raise awareness about food safety through a continuous promotional campaign focusing on topics such as hygienic food handling, food storage, and prevention of food poisoning. It can be seen that it is important to increase attitudes and intentions to purchase seafood products through proper knowledge of food safety.

The academic significance of this study is to verify the influences of various variables, such as demographics, socioeconomic characteristics, health-related characteristics, and safety awareness, by country of origin as factors affecting the level of concern about radioactivity in seafood. Specifically, this study investigated how consumers’ safety awareness of seafood in various countries affected their concerns about radioactivity, and the moderating effect of interest in food safety issues was identified. This can be seen as an original prior study that predicted the concern about radioactivity in seafood as an important factor that could affect the consumption of seafood and analyzed in depth the factors that could reduce it.

The practical implications of this study highlight the importance of increasing awareness of seafood safety. The seafood industry should invest in the production process to enhance the safety of seafood so that consumers can perceive it as safe. The government should apply new technologies to analyze seafood safety and radioactivity levels in seafood, which thus assures consumers’ seafood consumption. Furthermore, this study emphasizes the need for continuous education and promotional marketing in industry and government to increase interest in food safety issues.

## 5. Limitations

This study had some limitations. This study was initiated to reveal the impact of safety awareness regarding the country of origin on concerns about radioactivity and the moderating effect of interest in food safety issues among seafood-related food safety issues. A variety of seafood is sold, including domestic ones, as well as imported seafood, such as those from Japan, China, and Europe. Many factors can affect purchase attitudes or purchase intentions for the consumption of seafood, and in this study, concerns about radioactivity among food hazard factors were presented as a dependent variable. However, this research design may be an original study because there is little research that reveals the causal relationship between the awareness of safety by the country of origin of seafood and the level of concern about radioactivity in seafood, but it may be insufficient to provide a theoretical basis.

In addition, the representativeness of the sample is that a large proportion of elderly women were present because the sample was selected from those who mainly purchase food. In Korea, it seems possible to generalize the results, as women are the ones who mainly purchase food in the family. However, internationally, there may be differences by country, so it seems difficult to generalize internationally.

This study was a data-based quantitative study using secondary data related to consumers’ purchase of seafood. Although consumer awareness and attitudes are areas that can be explored in depth through qualitative analysis, this study mainly presented quantitative research, and although it presented intuitive results, there were limitations in presenting the results.

In future research, it will be necessary to utilize various variables to establish causal relationships between the independent and dependent variables presented in this study, and verifying them through theory or suggesting new theories may also be meaningful research. In particular, it appears that future research will be able to present research on the impact of the intention to purchase seafood targeting actual consumers by combining it with theory, rather than analyzing secondary data. In addition, it seems necessary to conduct research that reflects diverse population characteristics so that it can be applied not only in Korea but also internationally. In addition, it seems necessary to present results from a richer perspective through quantitative and qualitative research.

## 6. Conclusions

In this study, it was investigated how major food purchasers’ awareness of the safety of seafood by country of origin influenced their concerns about radioactivity, and the moderating effect of interest in food safety issues was examined. As a result, it was found that Korean consumers had a lower awareness of the safety of imported seafood compared with domestic seafood. Among the hazards of seafood, radioactivity was found to be the highest concern. Additionally, the lower the safety awareness of imported seafood, the higher the level of concern about radioactivity. However, interest in food safety issues was verified to have a moderating effect between the effect of awareness of the safety of European seafood among imported seafood and the level of radioactivity concern. In other words, in order to alleviate concerns about radioactivity in seafood, continuous monitoring and information disclosure to strengthen the safety awareness of imported seafood are important.

In addition, it is important to raise interest in food safety issues. Achieving this will require a joint effort from both industry stakeholders and government agencies.

This study was conducted on buyers who mainly purchase food and was intuitively connected to consumers’ food purchases. This will be a study that can be used not only in Korea but also internationally. This is because this study suggests that it is important to increase interest in food safety in order to improve the purchasing attitude and purchase intention of buyers who mainly purchase food in households. In order to provide correct food safety information, the population and culture are important. It can be seen that it is important to prepare a marketing strategy that takes into account the appropriate content and delivery method depending on the characteristics.

## Figures and Tables

**Table 1:** General demographic and socioeconomic characteristics of respondents (N = 3318).

Variable		% ^1^
Sex	Male	17.8
	Female	82.2
	Total	100.0
Age	20s	5.6
	30s	18.4
	40s	21.6
	50s	21.7
	60s and over	32.7
	Total	100.0
Education level	High school graduate or lower	58.6
	College graduate or higher	41.4
	Total	100.0
Occupation	Administrator/office work/professional	29.5
	Service	16.9
	Sales	14.4
	Agricultural and fisheries/simple labor	15.2
	Housewife	21.3
	Others	2.8
	Total	100.0
Monthly household income (KRW)	<2,000,000	21.3
	2,000,000–<3,000,000	20.9
	3,000,000–<4,000,000	15.8
	4,000,000–<5,000,000	13.0
	5,000,000–<6,000,000	13.5
	≥6,000,000	15.5
	Total	100.0
Number of household members	1	35.6
	2	27.7
	3	18.3
	≥4	18.4
	Total	100.0
Presence or absence of a young household member	Absence	86.8
	Presence	13.2
	Total	100.0
Administrative district	Dong	80.1
	Myon/Eup	19.9
	Total	100.0

Weighted analyses for complex sampling designs. ^1^ Values are presented as numbers (%).

**Table 2:** Health-related characteristics of respondents (N = 3318).

Variable	Mean ^1^ ± SE ^2^
Interest in health	3.77 ± 0.020
Current health status	3.62 ± 0.020

Weighted analyses for complex sampling designs. ^1^ A 5-point Likert scale ranging from 1 (very low) to 5 (very high) was used. ^2^ Standard error of the coefficient.

**Table 3:** Consumer behavior related to purchasing seafood (N = 3318).

Variable		% ^1^
Seafood purchasing criteria	Price	13.3
	Taste	29.4
	Safety	13.2
	Quality	29.6
	Nutrition (health)	4.3
	Convenience of purchase	4.5
	Convenience of cooking	5.7
	Total	100.0
Information to check when purchasing seafood	Price	11.5
	Place of production (West Coast, etc.)	11.6
	Country of origin (domestic, China, etc.)	15.7
	Freshness	47.4
	Brand (Chambada, Haneultteul, etc.)	1.3
	Appearance (shape, size, etc.)	6.4
	Whether it is a promotional product or not	1.9
	Type	2.2
	Packaging status	1.9
	Other certifications (HACCP ^2^, etc.)	0.1
	Whether functionality is added	0.1
	Total	100.0

Weighted analyses for complex sampling designs. ^1^ Values are presented as numbers (%). ^2^ HACCP: hazard analysis and critical control point.

**Table 4:** Frequency of purchasing seafood (N = 3318).

Variable	Mean ^1^ ± SE ^2^
Fisheries	3.55 ± 0.042
Seaweed (kelp, nori, etc.)	3.18 ± 0.042
Shellfish	2.63 ± 0.039
Mollusks (squid, octopus, etc.)	2.71 ± 0.035
Crustaceans (flower crab, etc.)	2.27 ± 0.029
Dried fish (anchovies, etc.)	2.89 ± 0.047

Weighted analyses for complex sampling designs. ^1^ A 7-point Likert-type scale ranging from 1 (very low) to 5 (very high) was used. 1: Do not eat/do not procure food; 2: less frequently; 3: once a month; 4: once a week; 5: rarely; 6: 2–3 times a week; and 7: daily. ^2^ SE: standard error of the coefficient.

**Table 5:** Safety awareness of seafood based on country of origin (N = 3318).

Variable	Mean ^1^ ± SE ^2^
Domestic (Korea)	3.90 ± 0.026
Japan	1.71 ± 0.033
China	1.78 ± 0.032
EU (European Union)	2.45 ± 0.031

Weighted analyses for complex sampling designs. ^1^ A 5-point Likert-type scale ranging from 1 (very low) to 5 (very high) was used. ^2^ SE: standard error of the coefficient.

**Table 6:** Concerns about hazards in seafood (N = 3318).

Variables	Mean ^1^ ± SE ^2^
Foreign substances	3.67 ± 0.036
Fish antibiotics	3.91 ± 0.035
Natural toxins (puffer fish, etc.)	3.88 ± 0.036
Heavy metals, environmental hormones, etc.	4.18 ± 0.033
Food poisoning bacteria	4.15 ± 0.034
Radioactivity	4.23 ± 0.037
Allergens	3.80 ± 0.032
Overall average of hazardous factors in seafood	4.09 ± 0.028

Weighted analyses for complex sampling designs. ^1^ A 5-point Likert-type scale ranging from 1 (very low) to 5 (very high) was used. ^2^ SE: standard error of the coefficient.

**Table 7:** Moderating effect of the interest in food safety issues on the relationship between safety awareness based on the country of origin of seafood and concern about radioactivity (N = 3318).

Variable		Model 1	Model 2	Model 3
		Β ^1^ (SE) ^2^	β (SE)	β (SE)
Sex ^3^	Male	−0.148 (0.094)	−0.145 (0.095)	−0.141 (0.095)
	Female	0.000	0.000	0.000
Age	20s	0.153 (0.157)	0.160 (0.154)	0.158 (0.153)
	30s	0.209 (0.098) *	0.217 (0.098) *	0.225 (0.099) *
	40s	0.118 (0.098)	0.122 (0.098)	0.122 (0.098)
	50s	0.164 (0.077) *	0.165 (0.077) *	0.161 (0.077) *
	60s and over	0.000	0.000	0.000
Educational level ^3^	High school graduate and lower	−0.082 (0.069)	−0.079 (0.068)	−0.072 (0.067)
	College graduate and higher	0.000	0.000	0.000
Occupation ^3^	Administrator/office work/professional	0.194 (0.175)	0.168 (0.173)	0.159 (0.174)
	Services	0.235 (0.172)	0.212 (0.170)	0.204 (0.169)
	Sales	0.242 (0.175)	0.223 (0.172)	0.211 (0.172)
	Agricultural and fisheries/simple labor	0.230 (0.183)	0.210 (0.180)	0.201 (0.180)
	Housewife	0.243 (0.170)	0.221 (0.166)	0.213 (0.166)
	Others	0.000	0.000	0.000
Monthly household income (KRW)	<2,000,000	0.149 (0.125)	0.143 (0.125)	0.136 (0.125)
	2,000,000–<3,000,000	0.250 (0.105) *	0.246 (0.105) *	0.239 (0.105) *
	3,000,000–<4,000,000	0.069 (0.104)	0.062 (0.104)	0.054 (0.104)
	4,000,000–<5,000,000	0.112 (0.080)	0.102 (0.081)	0.096 (0.081)
	5,000,000–<6,000,000	−0.024 (0.084)	−0.036 (0.083)	−0.036 (0.084)
	≥6,000,000	0.000	0.000	0.000
Number of household members	1	0.098 (0.135)	0.110 (0.135)	0.110 (0.133)
	2	0.118 (0.104)	0.127 (0.103)	0.128 (0.102)
	3	0.214 (0.087) *	0.217 (0.086) *	0.224 (0.086) *
	≥4	0.000	0.000	0.000
Presence or absence of a young household member ^3^	Absence	−0.020 (0.101)	−0.021 (0.101)	−0.014 (0.098)
	Presence	0.000	0.000	0.000
Administrative district ^3^	Dong	−0.056 (0.088)	−0.059 (0.088)	−0.052 (0.087)
	Myon/Eup	0.000	0.000	0.000
Interest in health		0.108 (0.028) ***	0.095 (0.028) **	0.089 (0.028) **
Current health status		−0.020 (0.029)	−0.022 (0.029)	−0.023 (0.029)
Safety awareness of domestic seafood ^4^		0.025 (0.031)	0.026 (0.031)	0.020 (0.031)
Safety awareness of Japanese seafood ^4^		−0.127 (0.040) **	−0.129 (0.040) **	−0.145 (0.041) ***
Safety awareness of Chinese seafood ^4^		−0.111 (0.046) *	−0.108 (0.047) *	−0.089 (0.048)
Safety awareness of European seafood ^4^		−0.054 (0.034)	−0.059 (0.034)	−0.068 (0.034) *
Interest in food safety issues			0.048 (0.031)	0.048 (0.031)
Safety awareness of domestic seafood × interest in food safety issues				0.001 (0.026)
Safety awareness of Japanese seafood × interest in food safety issues				0.051 (0.037)
Safety awareness of Chinese seafood × interest in food safety issues				−0.087 (0.045)
Safety awareness of European seafood × interest in food safety issues				0.077 (0.031) *
R^2^		0.094	0.096	0.101
Wald F (*p*)		3.279 (*p <* 0.001)	3.732 (*p <* 0.001)	4.179 (*p <* 0.001)

Weighted analyses for complex sampling designs. DV: concerns about radioactivity. ^1^ β: standardized regression coefficient. ^2^ SE: standard error of the coefficient. ^3^ Categorical variable. For each categorical variable, the bottom group is a reference group. ^4^ Continuous variables: Likert scale ranging from 1 (“not safe at all”) to 5 (“very safe”). * *p* < 0.05, ** *p* < 0.01, and *** *p* < 0.001.

## Data Availability

Korea Rural Economic Institute. 2021 Korean Consumer Behavior Survey for Food. Korea Rural Economic Institute: Naju, Korea, 2022. Available online at http://www.krei.re.kr/ (accessed on 5 December 2024).
